# A new role for concentrated solar radiation (CSR) as a renewable heat source for the catalyst-solvent free synthesis of tetrahydrobenzo[*b*]pyran scaffolds

**DOI:** 10.1038/s41598-023-38662-0

**Published:** 2023-07-17

**Authors:** Farzaneh Mohamadpour

**Affiliations:** School of Engineering, Apadana Institute of Higher Education, Shiraz, Iran

**Keywords:** Chemistry, Green chemistry, Renewable energy, Solar energy

## Abstract

Increased energy consumption as a result of population growth and industrialization necessitates the use of renewable energy sources in the field of chemistry. Nonrenewable energy sources release not only greenhouse gases but also other hazardous pollutants that are damaging to all living things. This plainly mandates the researchers' use of a renewable energy source that is both environmentally friendly and cost-effective. This study shows that a renewable energy source (sunlight) can be used to synthesize tetrahydrobenzo[*b*]pyran scaffolds using the Knoevenagel–Michael cyclocondensation of aldehyde derivatives, malononitrile, and dimedone via a three-condensation domino reaction. This research establishes a new role for solar energy as a renewable energy source for the synthesis of tetrahydrobenzo[*b*]pyran scaffolds under catalyst-solvent-free conditions, with outstanding yields, shorter reaction time, and great atom economy. This cyclization may also be done on a gram scale with free, safe, and clean energy from concentrated solar radiation (CSR), indicating the reaction's potential for industrial applications.

## Introduction

Nature has been directing humans on how to accomplish chemistry for a long time. We were unable to comprehend how nature, with its vast capacity, conducts intricate chemical processes in a biological system. Chemistry has relied on non-renewable resources in the past, as well as in the present, such as petroleum-based chemicals, which are commonly used as solvents. Apart from being non-renewable, these solvents are extremely harmful to both the environment and humans. Nonetheless, we have continued to use non-renewable materials in an irresponsible manner, despite the fact that the moment has come for the so-called "6th wave of innovation," in which sustainability is a top priority. As a result, many academic and industrial researchers are attempting to focus more on greener or more sustainable approaches to process development. In reality, most chemical and pharmaceutical companies now have a dedicated unit for environmentally friendly chemistry. Solvent, a medium required to accomplish chemical reactions, is a major difficulty, as is waste from chemical plants. Even a simple swap from organic to water as a solvent could drastically reduce the amount of organic solvent used and hence waste. When compared to organic solvents, one may argue that the expense of water over its life cycle or the treatment of wastewater is substantially higher. The amount of solvent required (water) is significantly smaller than that required for reactions carried out in organic solvents. This chemistry in water is swiftly progressing, however, it will be fascinating to see if the reaction is carried out neatly or without the need for a solvent, as "the best solvent is no solvent"^1^.

Because of the indiscriminate use of nonrenewable resources and the pressing need for sustainability, the time has come for the 6th wave of innovation, which entails the application of greener and more sustainable principles. In the development of processes, industries require us to consider a variety of factors such as the use of harmful chemicals, waste management, multistep processes, longer duration, reuse and recovery of materials, and so on. Given these considerations, a solvent- and catalyst-free process utilizing renewable energy sources is one of the most attractive approaches^2^.

**Figure 1:**
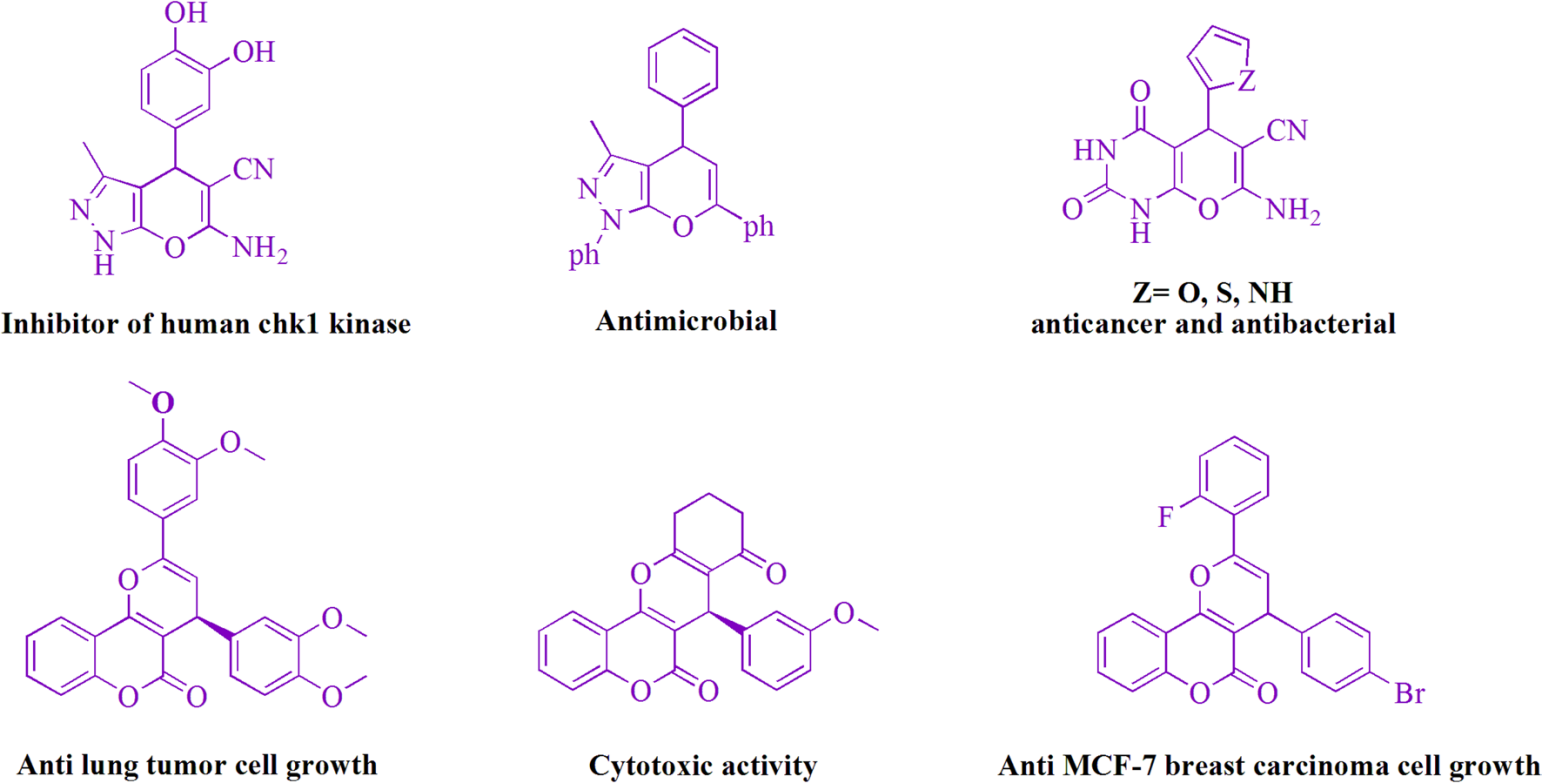
Pyran motifs are found in a number of medicinally significant substances.

Pyran derivatives with a variety of pharmacological properties (Fig. 1), including Chk1 kinase inhibitory activity^3^, analgesic properties^4^, anticancer^5^, vasodilatory activities^6^, spamolytic^7^, antihypertensive, hepatoprotective, cardiotonic^8^, vasodilator^9^, anti-leukemic^10,11^, emetic^12^, anti-anaphylactic activities^13^, diuretic^14^, and anti-alzheimer^15^.

These compounds can be synthesized in a variety of ways, employing a variety of catalysts like as.

CaHPO_4_^16^, SiO_2_NPs^17^, ethylenediamine diacetate^18^, SBPPSP^19^, I_2_^20^, NH_4_Al(SO_4_)_2_·12H_2_O^21^, NH_4_H_2_PO_4_/Al_2_O_3_^22^, ACoPc–MNPs^23^, ZnONPs^24^, Fe_3_O_4_@SiO_2_–imid–PMA^25^, NiFe_2_O_4_@SiO_2_–H_3_PW_12_O_40_^26^, theophylline^27^, triethanolamine^28^, NaN_3_^29^, Fe_3_O_4_@SiO_2_@TiO_2_^30^, MgFe_2_O_4_ nanoparticles^31^, trichloroisocyanuric acid^32^, Na_2_ eosin Y^33^, DABCO^34^, Pd nanoparticles^35^, WELPSA^36^, Core/Shell CaO@SiO_2_–SO_3_H^37^, Co_3_O_4_ nano-flakes^38^, HMS/Pr–Rh–Zr^39^, nano-SiO_2_/DBN^40^, M–Fe_3_O_4_@HAL–SO_3_H^41^, Bead-PIL^42^. It's worth noting that the Knoevenagel–Michael cyclocondensation reaction is a well-known method for producing tetrahydrobenzo[*b*]pyran scaffolds with either transition metal catalysts or organic solvents and/or conventional heating sources. Given the relevance of this reaction and our ongoing interest in green chemistry^43–46^, we predicted that this reaction could be performed without the use of a catalyst or solvent, as well as using just solar energy as a clean energy source. We are excited to show that this reaction might be carried out on a wider scale utilizing concentrated solar radiation (CSR), which is a free, safe, and clean energy source.

## Experimental

### General

The melting points of all compounds were determined using Electrothermal 9100 equipment. Furthermore, nuclear magnetic resonance (^1^HNMR, and ^13^CNMR) spectra were recorded using a Bruker DRX-400, Bruker DRX-300 and Bruker DRX-100 Avance instrument using CDCl_3_ as the solvent. The mass spectra were procured employing a spectrometer from Agilent Innovation (HP) working at a 70 eV ionization potential. The components (Carbon, Hydrogen, and Nitrogen) were examined employing a Heraeus CHN-O-Rapid analyzer. All reagents were purchased from Acros, Merck, and Fluka Chemicals and used without further purification.

### The entire procedure for preparing (4a–z)

The synthesis of tetrahydrobenzo[*b*]pyran derivatives was achieved using a three-condensation domino reaction involving aldehyde derivatives (**1**, 1.0 mmol), malononitrile (**2**, 1.0 mmol), and dimedone (**3**, 1.0 mmol) catalyzed by concentrated sun radiation (CSR) under catalyst-solvent free conditions (Fig. 6). For 2–6 min, the mixture was stirred. TLC was used to monitor the reaction process, with *n*-hexane/EtOAc (3:1) as the eluent. The achieved solid was filtered, and rinsed with water, and the crude solid was recrystallized from ethanol to yield pure material without the need for further purification. Although we were able to synthesize the above chemicals utilizing gram-scale processes, we wanted to test if it could be scaled up to the level that pharmaceutical process R&D prefers. Under CSR, 50 mmol each of m-tolualdehyde, malononitrile, and dimedone were agitated. As expected, the large-scale reaction occurred well and finished in 2 min, with the product collected by easy filtration as normal. This material's ^1^HNMR spectrum indicates that it is spectroscopically pure.

**Figure 2:**
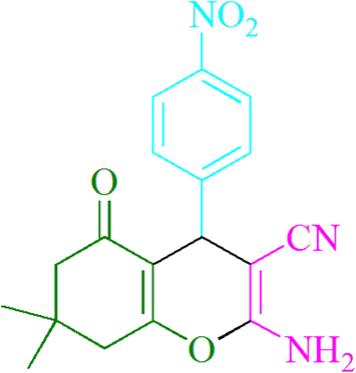
Structure for compound **4j.**

**Figure 3:**
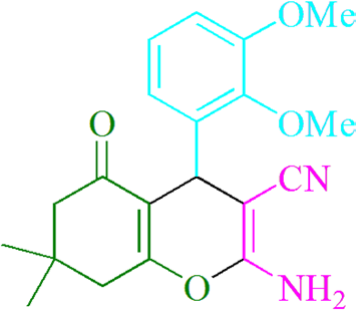
Structure for compound **4 s.**

**Figure 4:**
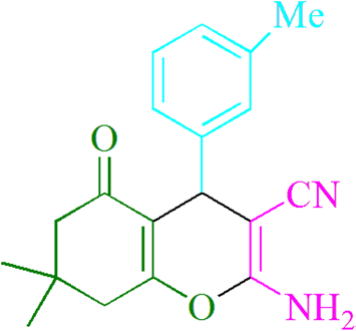
Structure for compound **4u.**

**Figure 5:**
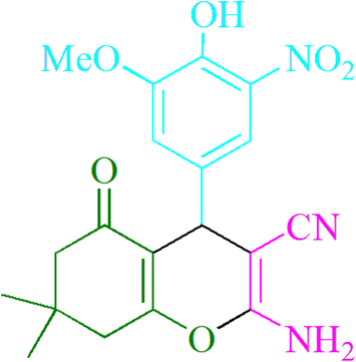
Structure for compound **4z.**

After comparing spectroscopic information, the products were categorized (^1^HNMR). The following sources and Figs. 2, 3, 4, and 5 provide support for this manuscript:

### 2-Amino-4-(4-nitrophenyl)-7,7-dimethyl-5-oxo-5,6,7,8-tetrahydro-4Hchromene-3-carbonitrile (4j)

Yield: 95%; M.p. 177–179 °C; ^1^HNMR (300 MHz, CDCl_3_) 1.07 (3H, s, CH_3_), 1.16 (3H, s, CH_3_), 2.30 (2H, d, *J* = 14.0 Hz, CH_2_), 2.52 (2H, s, CH_2_), 4.55 (1H, s, CHAr), 4.68 (2H, s, NH_2_), 7.45 (2H, d, *J* = 11.6 Hz, ArH), 8.20 (2H, d, *J* = 11.6 Hz, ArH).

### 2-Amino-4-(2,3-dimethoxyphenyl)-7,7-dimethyl-5-oxo-5,6,7,8-tetrahydro-4Hchromene-3-carbonitrile (4 s)

Yield: 92%; M.p. 218–220 °C; ^1^HNMR (300 MHz, CDCl_3_) 1.10 (3H, s, CH_3_), 1.14 (3H, s, CH_3_), 2.25 (2H, s, CH_2_), 2.47 (2H, s, CH_2_), 3.77 (3H, s, OCH_3_), 3.83 (3H, s, OCH_3_), 4.47 (2H, s, NH_2_), 4.73 (1H, s, CHAr), 6.68–6.84 (3H, m, ArH).

### 2-Amino-4-(3-methylphenyl)-7,7-dimethyl-5-oxo-5,6,7,8-tetrahydro-4Hchromene-3-carbonitrile (4u)

Yield: 96%; M.p. 199–201 °C; ^1^HNMR (400 MHz, CDCl_3_) 1.06 (3H, s, CH_3_), 1.13 (3H, s, CH_3_), 2.23 (2H, d, *J* = 5.6 Hz, CH_2_), 2.31 (3H, s, CH_3_), 2.46 (2H, s, CH_2_), 4.38 (1H, s, CHAr), 4.52 (2H, s, NH_2_), 7.09–7.15 (3H, m, ArH), 7.28 (1H, s, ArH).

### 2-Amino-4-(3-nitro-4-hydroxy-5-methoxyphenyl)-7,7-dimethyl-5-oxo-5,6,7,8-tetrahydro-4Hchromene-3-carbonitrile (4z)

Yield: 89%; M.p. 222–224 °C; ^1^HNMR (400 MHz, CDCl_3_) 1.08 (3H, s, CH_3_), 1.15 (3H, s, CH_3_), 2.27 (2H, d, *J* = 5.6 Hz, CH_2_), 2.50 (2H, s, CH_2_), 3.99 (3H, s, OCH_3_), 4.41 (1H, s, CHAr), 4.68 (2H, s, NH_2_), 7.19–7.47 (2H, m, ArH), 10.68 (1H, s, OH); ^13^CNMR (100 MHz, CDCl_3_): 27.5, 28.9, 32.2, 35.2, 40.6, 50.6, 56.8, 62.2, 114.0, 118.3, 134.9, 137.2, 145.5, 149.0, 149.8, 154.9, 157.6, 162.1, 195.9; Anal. Calcd for C_19_H_19_N_3_O_6_: C, 59.22; H, 4.97; N, 10.90%. Found: C, 59.29; H, 4.86; N, 10.98%; MS (*m/z*): 386 (*M* +) (Supplementary Fig. S1).

## Results and discussion

**Figure 6:**
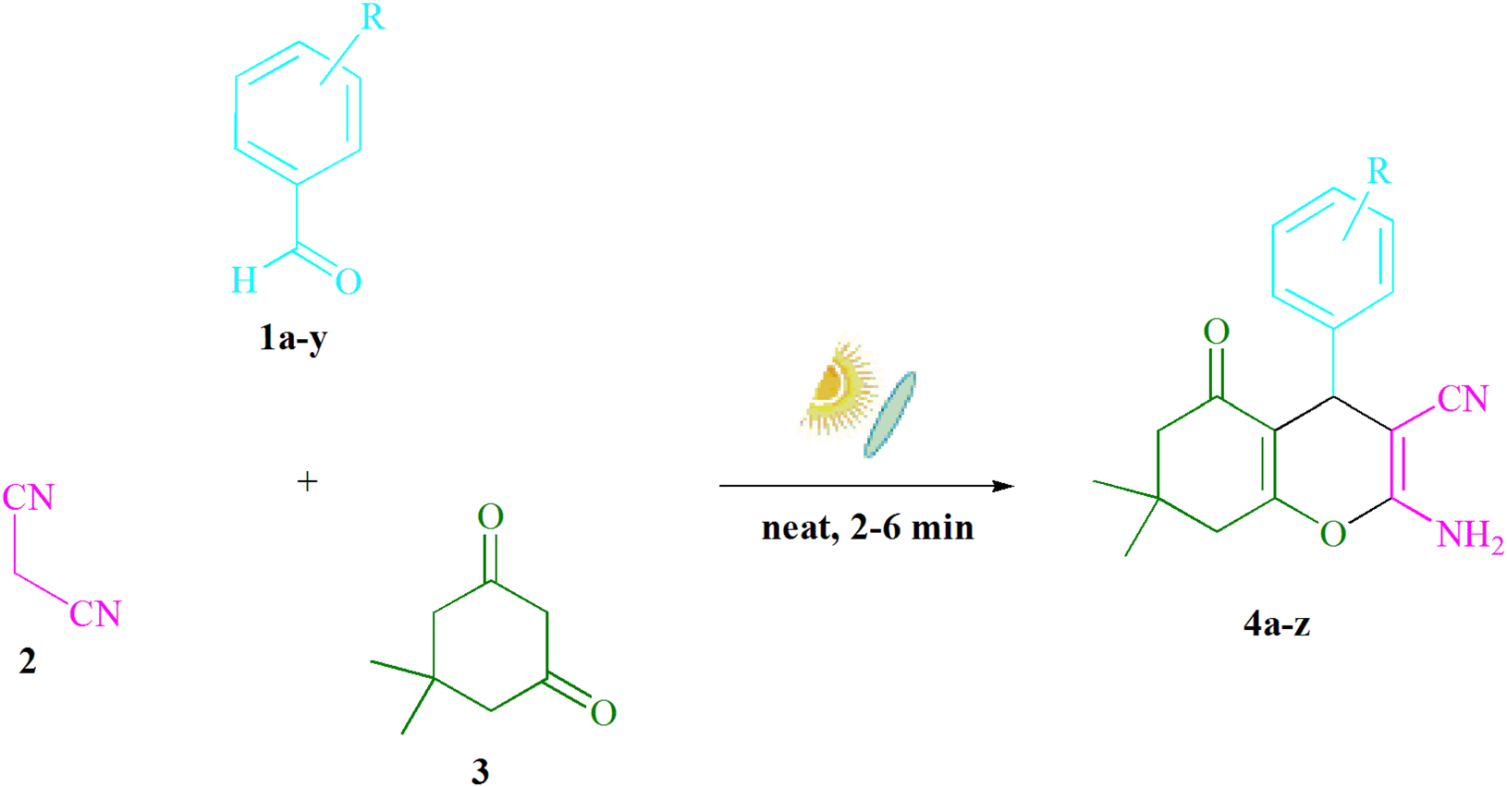
Tetrahydrobenzo[*b*]pyran scaffold synthesis.

CSR was used to stir benzaldehyde (1 mmol), malononitrile (1 mmol), and dimedone (1 mmol) in EtOH for 5 min, yielding 85% of the desired result (Table 1, entry 1). Furthermore, conducting the reaction in H_2_O, MeOH, DMSO, DMF, THF, CH_3_CN, or EtOAc did not result in a higher chemical yield (Table 1). We eventually tried heating these with CSR by simply combining them. Surprisingly, the reaction was completed in under 3 min, yielding the quantitatively desired result (Table 1, entry 3). It's only natural to point out that recrystallization from ethanol yielded spectroscopically pure product **4a**. Because no column chromatography was necessary, this clearly indicates that the new approach is far superior in terms of product isolation. CSR set-up was used to synthesize tetrahydrobenzo[*b*]pyran scaffolds according to the published procedures^1,2^. With the right circumstances in place, a wide range of substrates were investigated (Table 2 and Fig. 6). It's worth mentioning that the benzaldehyde substituent had no effect on the reaction's outcome (Table 2). The reaction conditions were tolerant of polar and halide substitutions. At the current reaction state, reactions with both electron-donating and electron-withdrawing functional groups went smoothly. The yield of all *ortho*, *meta*, and *para*-substituted aromatic aldehydes is exceptionally high. Different aldehydes, such as the bulkier naphthaldehyde, provide a finished product with minimal yield loss. In terms of reactivity, heterocyclic aldehydes followed a similar pattern (Table 2).

**Table 1:** The optimization table for the **4a** synthesis^*a*^.


Entry	Solvent	Time (min)	Isolated yields (%)
1	EtOH	5	85
2	H_2_O	10	47
**3**	**–**	**3**	**96**
4	MeOH	10	61
5	DMSO	25	23
6	DMF	25	18
7	THF	25	27
8	CH_3_CN	15	21
9	EtOAc	20	16

^a^Reaction conditions: In the solvents (3 mL) indicated, benzaldehyde (1 mmol), malononitrile (1 mmol), and dimedone (1 mmol) were agitated under CSR.

Significant values are in bold.

**Table 2:** Substrate scope for tetrahydrobenzo[*b*]pyran scaffolds.


**4a** (3 min, 96%) Mp. 224-226 °C Lit. 226-228 °C^16^	**4b** (2 min, 97%) Mp. 222-224 °C Lit. 223-226 °C^16^	4c (2 min, 94%) Mp. 220-222 °C Lit. 221-223 °C^19^	**4d** (4 min, 91%) Mp. 228-230 °C Lit. 227-229 °C^24^
**4e** (2 min, 98%) Mp. 210-212 °C Lit. 210-212 °C^30^	**4f** (4 min, 90%) Mp. 213-215 °C Lit. 214-216 °C^18^	**4g** (2 min, 93%) Mp. 210-212 °C Lit. 208-210 °C^26^	**4h** (2 min, 96%) Mp. 214-216 °C Lit. 216-217 °C^26^
**4i** (5 min, 88%) Mp. 229-231 °C Lit. 227-229 °C^24^	**4j** (3 min, 95%) Mp. 177-179 °C Lit. 180-181 °C^18^	**4k** (4 min, 91%) Mp. 209-211 °C Lit. 208-210 °C^18^	**4l** (6 min, 88%) Mp. 227-229 °C Lit. 228-230 °C^17^
**4m** (5 min, 91%) Mp. 216-218 °C Lit. 215-218 °C^30^	**4n** (6 min, 86%) Mp. 206-208 °C Lit. 204-206 °C^16^	**4o** (5 min, 85%) Mp. 224-226 °C Lit. 226-228 °C^23^	**4p** (3 min, 93%) Mp. 200-202 °C Lit. 202-205 °C^16^
**4q** (2 min, 97%) Mp. 212-214 °C Lit. 211-212 °C^26^	**4r** (6 min, 87%) Mp. 208-210 °C Lit. 210-212 °C^23^	**4s** (4 min, 92%) Mp. 218-220 °C Lit. 217-219 °C^17^	**4t **(2 min, 97%) Mp. 200-202 °C Lit. 198-200 °C^19^
**4u** (2 min, 96%) Mp. 199-201 °C Lit. 198-200 °C^19^	**4v** (4 min, 83%) Mp. 148-150 °C Lit. 150-152 °C^30^	**4w** (3 min, 96%) Mp. 211-213 °C Lit. 211-212 °C^26^	**4x** (5 min, 87%) Mp. 229-231 °C Lit. 228-230 °C^16^
**4y** (3 min, 93%) Mp. 208-210 °C Lit. 210-212 °C^32^	**4z** (5 min, 89%) Mp. 222-224 °C^a^		

^*a*^The new compound is synthesized in this work.

**Figure 7:**
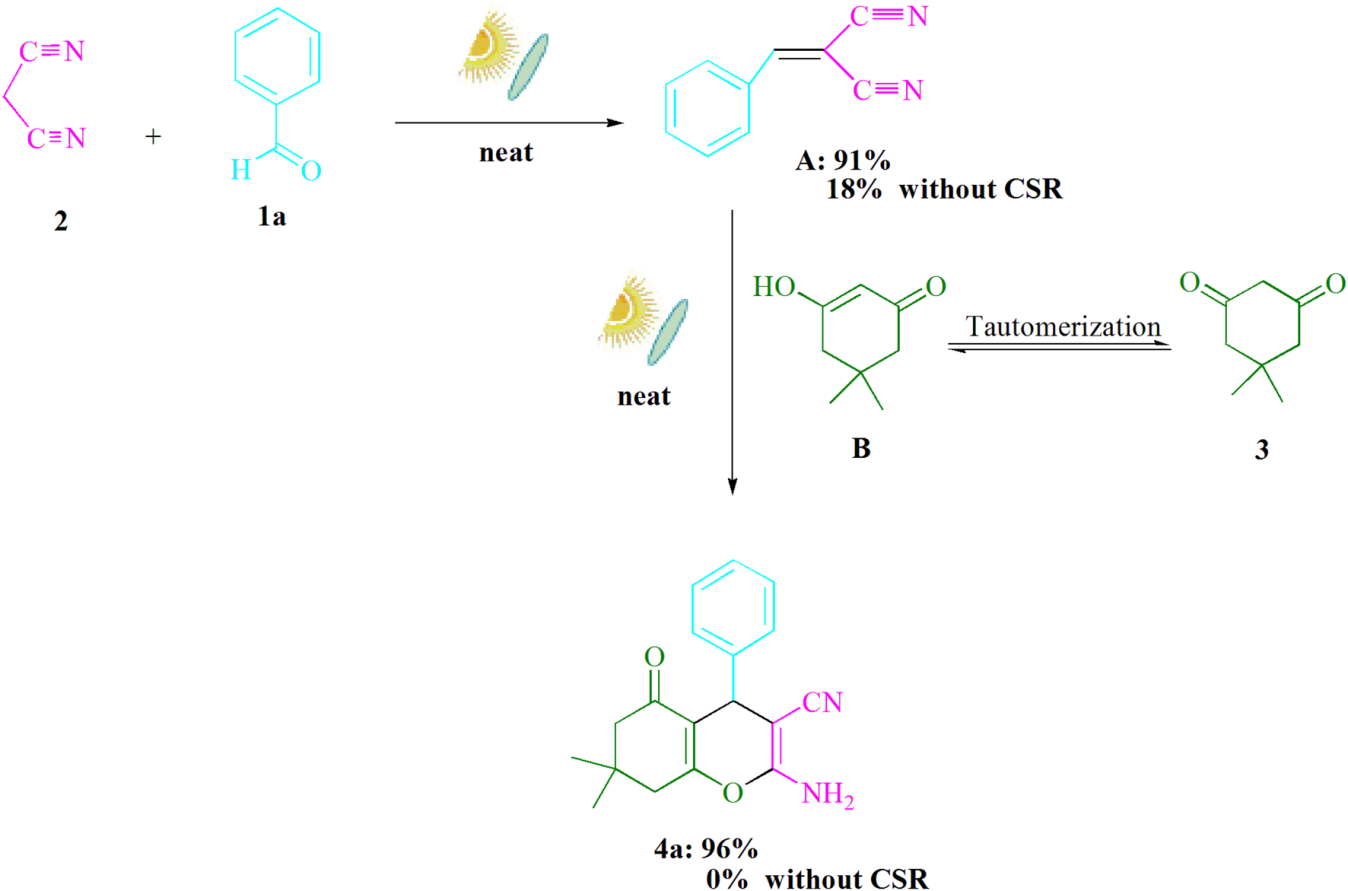
Control experiments on intermediates.

Experiments on intermediates have also been studied as a control. The Knoevenagel-Michael cyclocondensation reaction has two steps: the first is the production of arylidenemalononitrile **A**, and the second is the condensation of **A** with **B**. Separate processes were done to extract intermediates in order to determine which step actually needed a solar heat source (Fig. 7). Under sun radiation, benzaldehyde (**1a**) and malononitrile (**2**) were first agitated. In 91% of cases, the intermediate product (**A**) was separated. Only 18% yield of the intended product **A** was produced when the reaction was performed without CSR but in refluxing ethanol. In addition, no desirable product **4a** was obtained when **A** was refluxed in ethanol with dimedone. When **A** was mixed with dimedone in the presence of CSR, however, the desired product **4a** was formed 96% of the time. According to the reported methods^1,2^, we conducted several control experiments (Fig. 7) to determine which step actually requires solar radiation. We discovered that both steps in Fig. 7 required CSR because they did not give any significant conversion at room temperature or conventional heating at 90–100 °C. UV–visible and infrared light are both present in solar radiation. UV radiation's photocatalytic characteristic, along with highly rapid vibrational movements of bonds (IR irradiation), causes a rapid collision of reactants, resulting in a chemical transformation in a short period of time. The enhancement of the chemical reaction rate could be due to the synergistic effect of UV and IR light.

Table 3 shows a comparison of the catalytic capability of a number of catalysts mentioned in this study for the manufacture of tetrahydrobenzo[*b*]pyran scaffolds. It could be used in a variety of ways, including the utilization of a renewable energy source (sunlight), catalyst-solvent-free conditions with high yields, and time-saving elements of the reaction. On a multigram scale, the atom-economic protocol is effective and has substantial industrial applications. These goods excel in terms of both performance and purity.

**Table 3:** Catalytic ability of some of the catalysts in the manuscript for the production of tetrahydrobenzo[*b*]pyran scaffolds^*a*^.

Entry	Catalyst	Conditions	Time/yield (%) references
1	WELPSA	H_2_O, Microwave, 80 °C	10 h/88^36^
2	Core/Shell CaO@SiO_2_-SO_3_H	H_2_O, 50 °C	20 min/93^37^
3	Co_3_O_4_ nano-flakes	EtOH/H_2_O, rt	5 min/95^38^
4	HMS/Pr-Rh-Zr	PEG, 80 °C	30 min/87^39^
5	nano-SiO_2_/DBN	H_2_O/EtOH, 60 °C	20 min/85^40^
6	M-Fe_3_O_4_@HAL-SO_3_H	H_2_O/EtOH, 80 °C	1.5 h/95^41^
7	Bead-PIL	H_2_O, rt	40 min/97^42^
**8**	**Catalyst-solvent free conditions**	**Concentrated solar radiation (CSR)**	**3 min/96 (this work)**

Significant values are in bold

^*a*^Based on the three-component reaction of benzaldehyde, malononitrile, and dimedone.

## Conclusion

To conclude, a very efficient and long-lasting synthesis of tetrahydrobenzo[*b*]pyran scaffolds has been discovered via a Knoevenagel–Michael cyclocondensation reaction mediated by concentrated sun radiation (CSR). This reaction has been carried out on a variety of substrates using a renewable energy source (sunlight) and a relatively simple experimental apparatus. The reaction was highly quick and did not necessitate the use of a solvent or chromatographic purification. This reaction might be scaled up without sacrificing the outcome, according to a multigram scale reaction of model substrates. Furthermore, the use of this approach to synthesize real-world medicinal compounds demonstrated its broad applicability.

## Supplementary Information

Supplementary Figures.

## Data Availability

All data generated or analyzed during this study are included in this published article [and its supplementary information files].
