# Supplementary material for: A new role for concentrated solar radiation (CSR) as a renewable heat source for the catalyst-solvent free synthesis of tetrahydrobenzo[b]pyran scaffolds
Source: Sci Rep. 2023 Jul 17;13:11485. doi: 10.1038/s41598-023-38662-0 (PMC10352380; doi:10.1038/s41598-023-38662-0)
Supplement: Supplementary file 1 — Supplementary Figures. [file 41598_2023_38662_MOESM1_ESM.pdf]

Supporting Information

A new role for concentrated solar radiation (CSR) as a renewable heat source for the catalyst-solvent free synthesis of tetrahydrobenzo[*b*]pyran scaffolds

Farzaneh Mohamadpour *

School of Engineering, Apadana Institute of Higher Education, Shiraz, Iran

* Corresponding author. mohamadpour.f.7@gmail.com

2-Amino-4-(3-nitro-4-hydroxy-5-methoxyphenyl)-7,7-dimethyl-5-oxo-5,6,7,8-tetrahydro-4Hchromene-3-carbonitrile (4z)

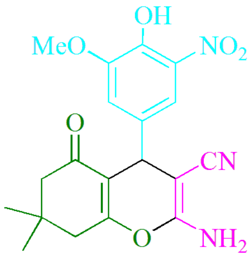

Figure S1. Structure for compound **4z**

Yield: 89%; M.p. 222-224 °C; ^{1}HNMR (400MHz, $CDCl_3$) 1.08 (3H, s, CH_3), 1.15 (3H, s, CH_3), 2.27 (2H, d, *J*=5.6 Hz, CH_2), 2.50 (2H, s, CH_2), 3.99 (3H, s, OCH_3), 4.41 (1H, s, CHAr), 4.68 (2H, s, NH_2), 7.19-7.47 (2H, m, ArH), 10.68 (1H, s, OH); ^{13}CNMR (100 MHz, $CDCl_3$): 27.5, 28.9, 32.2, 35.2, 40.6, 50.6, 56.8, 62.2, 114.0, 118.3, 134.9, 137.2, 145.5, 149.0, 149.8, 154.9, 157.6, 162.1, 195.9; Anal. Calcd for $C_{19}H_{19}N_3O_6$: C, 59.22; H, 4.97; N, 10.90%. Found: C, 59.29; H, 4.86; N, 10.98%; MS (*m/z*): 386 (*M* +).

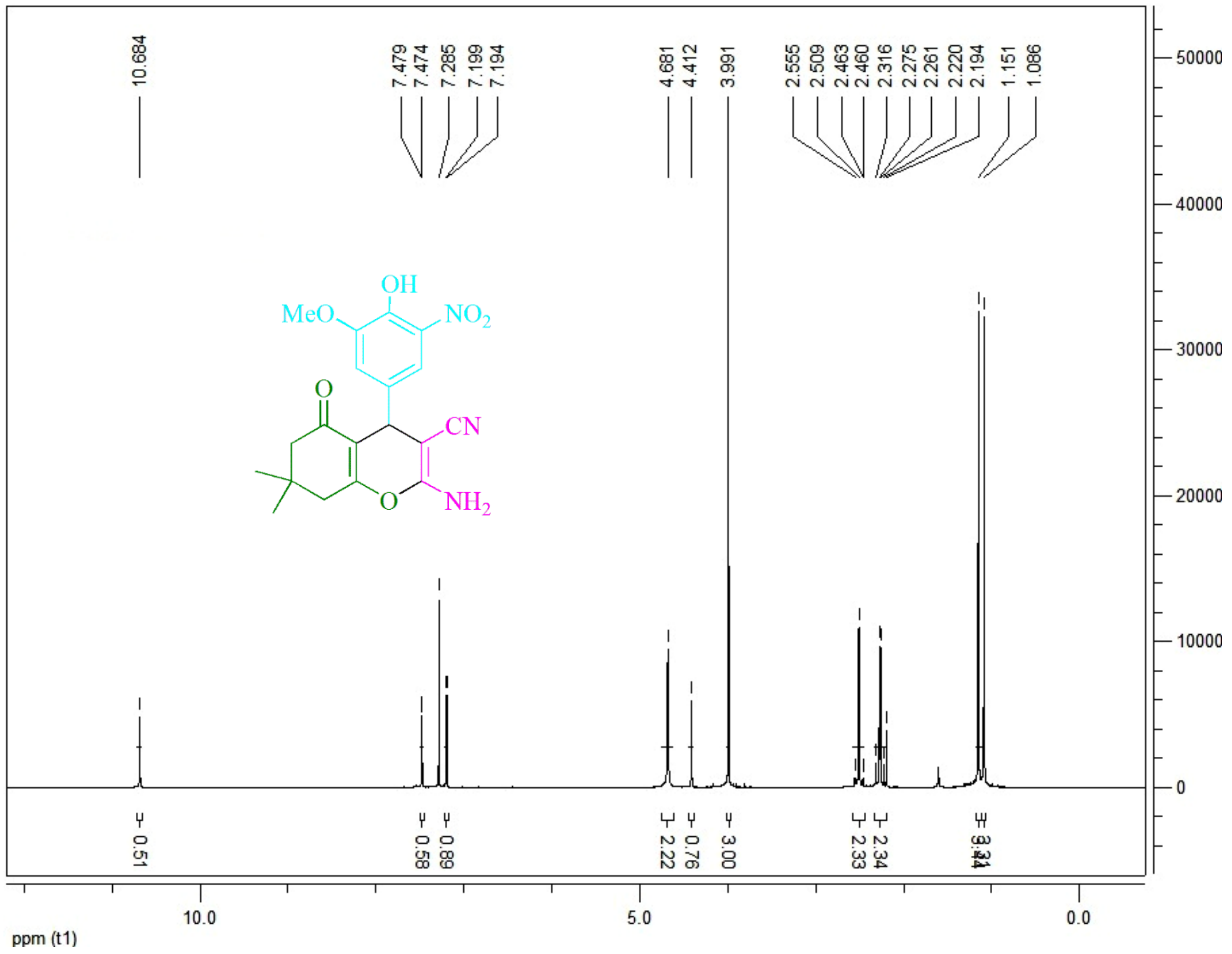

Figure S2.^{1}HNMR Spectrum of compound (400 MHz, $CDCl_3$) of **4z**

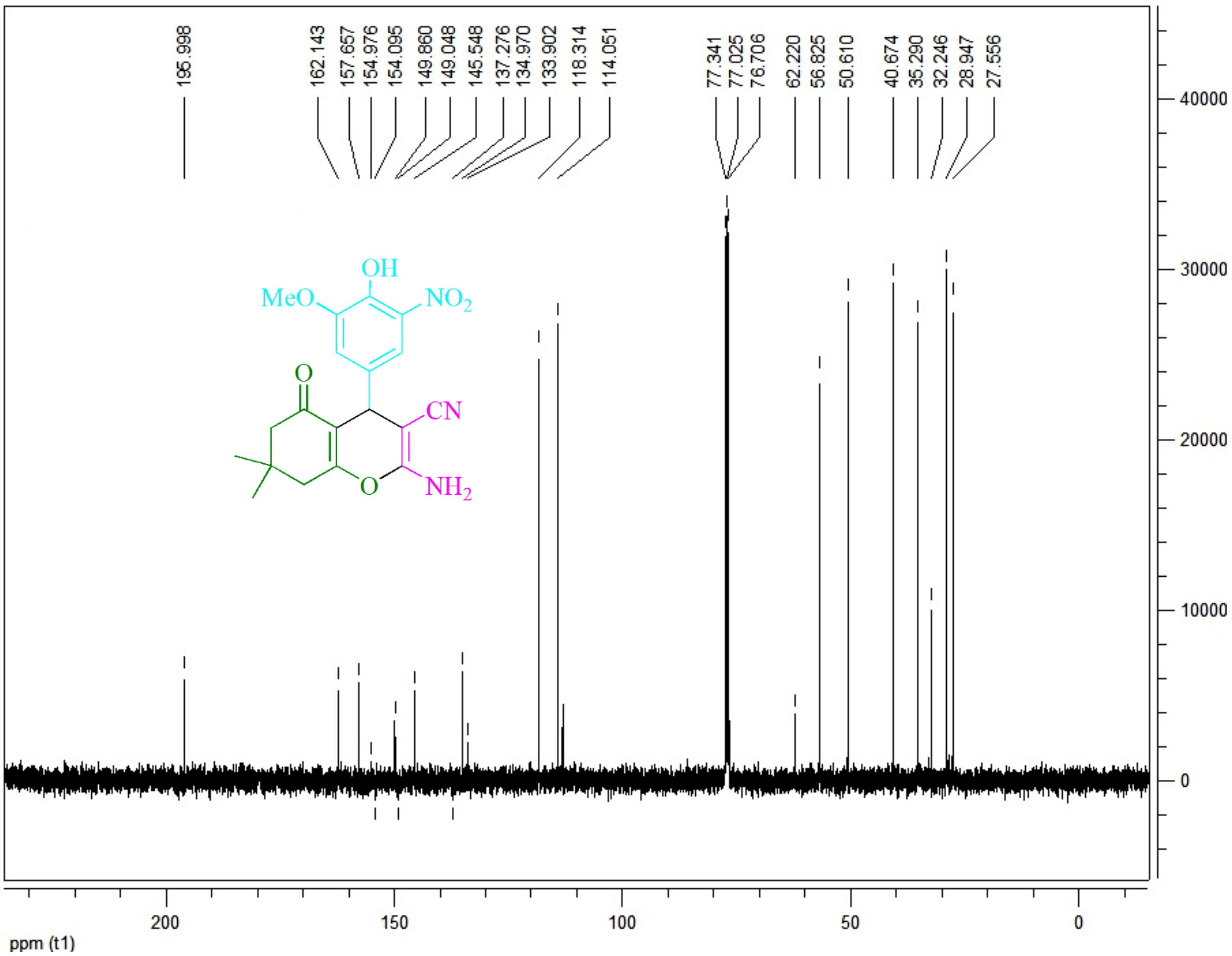

Figure S3. ^{13}CNMR Spectrum of compound (100 MHz, $CDCl_3$) of **4z**
